# Atypical cerebral lateralisation in adults with compensated developmental dyslexia demonstrated using functional transcranial Doppler ultrasound

**DOI:** 10.1016/j.bandl.2009.05.002

**Published:** 2009-10

**Authors:** Sarah Illingworth, Dorothy V.M. Bishop

**Affiliations:** University of Oxford, Department of Experimental Psychology, South Parks Road, OX1 3UD, United Kingdom

**Keywords:** Cerebral lateralisation, Dyslexia, Functional transcranial Doppler ultrasound

## Abstract

Functional transcranial Doppler ultrasound (fTCD) is a relatively new and non-invasive technique that assesses cerebral lateralisation through measurements of blood flow velocity in the middle cerebral arteries. In this study fTCD was used to compare functional asymmetry during a word generation task between a group of 30 dyslexic adults and a group of 30 non-dyslexic individuals. In light of previous evidence of atypical laterality in dyslexia, a reduced leftward asymmetry was predicted and confirmed. We know from previous research that most people with atypical language lateralisation have normal language and literacy skills: nevertheless, our results confirm that language laterality is reduced in those with dyslexia. Theoretical explanations for this apparent conundrum are discussed.

## 1 Introduction

The diagnosis of developmental dyslexia is made when a person’s literacy skills are poor in relation to other cognitive abilities for no obvious reason. Dyslexia is typically characterised by phonological deficits, and is thought to be neurobiological in origin (Lyon, 2003). A postulated link with laterality dates back to Orton (1925), who proposed delayed neurological development leading to a lack of a dominant hemisphere as the cause of developmental dyslexia.

Orton’s original speculations were based on observations of mirror-image confusions in letter-writing and atypical handedness in poor readers. However, this evidence is not at all compelling. First, mirror-image confusions have been shown to be a characteristic of typical children at early stages of learning to read, and cannot be regarded as indicative of cerebral abnormality (Liberman, Shankweiler, Orlando, Harris, & Berti, 1971). Second, handedness is only a weak indicator of cerebral language lateralisation, with over 90% of right-handers and around 70–80% of left-handers having left-hemisphere language (Josse & Tzourio-Mazoyer, 2004). Furthermore, an association between handedness and reading difficulties is not consistently found, and the failure to confirm it in large-scale studies suggests that the ‘file-drawer problem’ may be an issue here: handedness is so easy to measure, that it tends to be included in a study but then reported only when significant associations are found (Bishop, 1990a). This problem is compounded by the fact that there is no agreement as to how handedness should be categorised, giving considerable potential for post hoc groupings to be formed (Bishop, 1990b).

A more direct behavioural measurement of cerebral lateralisation for language is provided by dichotic listening tasks, in which competing stimuli are simultaneously presented to each ear. Atypical laterality on dichotic listening has been found in people with poor literacy skills, but a consistent performance profile is missing: in a review, Bryden (1988) found that 14 studies found no evidence of difference between poor readers and controls, 30 showed poor readers to be less lateralised, and 7 found poor readers to be more strongly lateralised. A limitation of the dichotic listening method is that, though it reliably shows left-hemisphere advantage for language at the group level, it has relatively low test–retest reliability in individuals (Voyer, 1998).

Inconsistency of findings also characterises the literature on structural brain asymmetry in dyslexia. Leonard and Eckert (2008) suggested that some of this variation may be related to phenotypic heterogeneity: they reviewed their own studies, which found that symmetrical brains were seen in individuals with poor language comprehension, whereas the opposite pattern, an enhancement of the normal leftward asymmetry, characterised poor readers with more circumscribed deficits in phonological processing. However, the extent to which structural asymmetry in dyslexia predicts functional asymmetry remains unclear.

With the advent of functional imaging, it became possible to look directly at brain activation during language tasks. In a review of fMRI and PET studies, Temple (2002) concluded that there was a general trend of reduced activity in left hemisphere temporo-parietal cortex in people with dyslexia. It can be difficult, however, to know how far this is a consequence rather than a cause of poor reading ability, since the tasks used to activate the brain typically involve written language. Also, as with studies on brain structure, there is suggestive evidence that the specific phenotype may be important. Shaywitz et al. (2003) compared adults with persistent reading difficulties, compensated poor readers, and normal readers. Whereas persistent poor readers showed normal activation of the posterior reading circuits, compensated poor readers showed relative underactivation of left-hemisphere reading circuits coupled with enhanced right hemisphere activation. This was interpreted as compensatory activity, although it could be indicative of atypical cerebral lateralisation that had been present from the outset of learning to read.

In recent years, functional transcranial Doppler ultrasonography has been used as a non-invasive and inexpensive alternative way of measuring cognitively induced changes in cerebral blood flow velocity in the middle cerebral arteries (MCA). Typically, cerebral lateralisation has been assessed using a word generation task (Knecht et al., 1998). The participant is shown a letter and asked to silently generate as many words as possible beginning with that letter, allowing investigation of the neural correlates of language generation without the possibility of motor artefacts associated with speech. Deppe, Knecht, Henningsen, and Ringelstein (1997) developed an analytic method (Average), which takes out the effect of the heart rate cycle, and adjusts for differences in overall blood flow between left and right sides (Deppe et al., 1997). A laterality index is computed as mean amplitude around a peak in the difference waveform between standardised left and right MCA blood flow velocity. When the Average software is used to process the data, the correlation between odd and even epoch estimates of the laterality index in a test session is typically around .8 or higher (Bishop, Watt, & Papadatou-Pastou, 2009; Lohmann, Drager, Muller-Ehrenberg, Deppe, & Knecht, 2005), giving confidence that this method is reliable at the individual level. Although it has poor spatial resolution, and involves a different approach to quantifying laterality (Seghier, 2008), laterality indices from a word generation task agree well with those obtained using fMRI (Deppe et al., 2000).

The principal limitations of functional TCD are, first, that it has very poor spatial resolution and cannot be used to localise activity within the territories of the middle cerebral artery, and second, that it is unsuitable in approximately 5% of participants who lack a temporal window, i.e. the region in front of the ear where the skull is thin enough to allow for penetration by the ultrasonic beam.

In the current study, we assessed handedness and cerebral lateralisation using the standard word generation task with fTCD. We focused on compensated adult dyslexics of high ability to ensure that literacy skills were sufficient for the word generation task, and we also explored the correlation between handedness, degree of language lateralisation, and number of words generated. Participants were 30 dyslexic (19 female, 11 male) and 30 non-dyslexic (21 female, 9 male) native English-speaking adults. Participants with dyslexia had to demonstrate deficits on a standardised dyslexia assessment (see below) in the context of a prior diagnosis of dyslexia by an educational psychologist. Most participants were Oxford University students and staff, recruited through advertisements and emails to the colleges and departments of the university.

## 2 Results

Table 1 shows the characteristics of the two participant groups on the behavioural tests. As expected given the inclusion criteria, the dyslexic group scored significantly lower on the average YAA score, *F*(1, 58) = 53.8, *p* < .001. The two groups had been matched on age and nonverbal ability, and they did not differ significantly on handedness: *F*(1, 58) = 1.8, *p* = .176. Three of the non-dyslexic and five of the dyslexic participants had a handedness quotient at or below zero, denoting left-handedness.

**Fig. 1:**
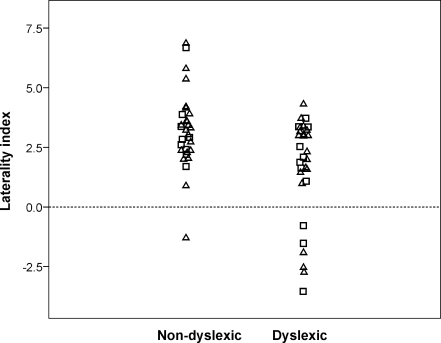
Scatterplot showing distribution of laterality indices on word generation task for non-dyslexic and dyslexic males (squares) and females (triangles).

Fig. 1 shows a scatterplot of the laterality indices (LI) for the word generation paradigm for non-dyslexic and dyslexic groups. Inspection of the figure suggests that dyslexics (*M* = 1.65, *SD* = 2.15) show less left lateralisation than non-dyslexics (*M* *=* 3.19, *SD* = 1.61). Because of skew in the data, we also considered degree of overlap between groups, and found that 73% of the dyslexics scored below the control mean; for normally distributed data this would correspond to an effect size (Cohen’s *d*) of 0.6. A Mann–Whitney test was used to test the significance of the difference; *U* = 267, *p* (2-tailed) = .007. In addition, the standard error of each participant’s LI was used to compute a 95% confidence interval to determine if it differed significantly from zero. This showed that of the non-dyslexics, 28 were left-lateralised, none was right-lateralised and two were bilateral (i.e. the LI was not significantly different from zero). For the participants with dyslexia, 23 were left-lateralised, four were right-lateralised and three were bilateral. The difference in frequency of left-lateralised cases between the dyslexic and non-dyslexic groups just fell short of statistical significance, Fisher exact test, *p* = .073. As can be seen from the scatter-plot, the quantitative analysis appears more sensitive to the group difference because the dyslexic distribution is shifted from leftward bias across the range, leading not only to more bilateral and right-lateralised cases, but also to fewer cases of strong left lateralisation. In addition, the correlation between the handedness quotient and the LI on fTCD was close to zero, *r*(60) = .06, *p* = .667.

Although the smaller sample size gave reduced power to detect effects, especially in males (*N* = 11 non-dyslexic and 9 dyslexic), it was noteworthy that the same pattern of results was seen when the sample was subdivided by gender. For males, the mean LI for non-dyslexics was 3.17 (*SD* = 1.46) vs. 1.25 (*SD* = 2.29) for dyslexics, Mann–Whitney *U* = 24, *p* (2-tailed) = .053; for females the mean LI for non-dyslexics was 3.20 (*SD* = 1.7) vs. 1.89 (*SD* = 2.09) for dyslexics, Mann–Whitney *U* = 121, *p* (2-tailed) = .033.

One concern is that the reduced left lateralisation in the dyslexic group could simply reflect poor ability on the word generation task. If the dyslexics are less able to generate words, then they may engage left-hemisphere systems less strongly. There was a small but significant difference between groups in the mean number of words generated per letter: for non-dyslexics, *M* = 4.34, *SD* = .602; for dyslexics, *M* = 3.98, *SD* = .582, *t*(58) = 2.33, *p* = .023. However, performance on the word generation task was unrelated to the LI from fTCD, *r*(60) = .04, *p* = .791.

A further possibility is that the LI computed from the word generation task is simply less reliable in the dyslexic group. Split half reliability for word generation LIs was computed from Pearson correlations for the LIs from odd and even epochs. For the whole group, *r* = .72, *p* < .001, for the 30 non-dyslexics, *r* = .44, *p* = .015, and for the 30 dyslexics, *r* = .84, *p* < .001. It is clear that the reduced lateralisation in the dyslexic group is not the consequence of unreliability of the LI estimates. The reliability for the non-dyslexic group, though significant, was lower than is usually found, but this could be because this group contained a high proportion of cases with left-hemisphere speech, leading to restriction of range.

## 3 Discussion

This study found that, as predicted, the dyslexic group showed significantly less left-lateralisation than the non-dyslexic group on the word generation task. The significant difference in LI between groups represented a shift downwards in the distribution for individuals with dyslexia, rather than an increase in the number of right-lateralised individuals. Categorisation into qualitative laterality groups showed that majority of those with dyslexia were left-lateralised, with only four being significantly right-lateralised. In contrast, two of the non-dyslexic participants showed bilateral activation, none was found to be significantly right-lateralised. This is rather unusual in a group of this size. In a previous investigation of language lateralisation using the word generation fTCD paradigm, 7.5% of 188 healthy right-handed subjects were found to have right hemisphere dominance (Knecht et al., 2000). This would predict that approximately two of the normal participants in this study would be expected to show right hemisphere dominance. The absence of right-lateralised individuals in the non-dyslexic group is therefore likely to reflect sampling error. Despite this, the higher value of the left-lateralised LIs in the non-dyslexic group compared to those in the dyslexic group, and the moderate effect size, suggest this finding is reliable. As a further check, two of the left-lateralised non-dyslexic cases were selected at random and given LI scores of opposite sign (i.e. right-lateralised), and the comparison was repeated. The group difference remained statistically significant on this more stringent test (*p* = .017).

The relatively high proportion of females in our dyslexic sample requires some comment, given that dyslexia is usually thought to be commoner in males (Rutter et al., 2004). We suggest that two factors contributed to the female excess. First, students who can cope with a rigorous academic environment despite dyslexia are likely to be those with milder difficulties, who are more likely to be female (Feldman et al., 1995). In addition, there is some evidence of female bias in volunteers for research studies (e.g., Lykken, Tellegen, & DeRubeis, 1978), an effect that may be compounded if the focus is on the individual’s impairment.

We are aware of only one previous study in which fTCD was used with individuals with literacy difficulties, by Whitehouse and Bishop (2008). They found an unusually high rate of abnormal language laterality in poor readers, with a high frequency of both right-hemisphere and bilateral language. Their sample, however, was more severely impaired and had a history of developmental language impairment, with nonverbal ability at the lower end of the normal range. Intriguingly, another group with a history of language difficulties who were reading within the normal range had normal language lateralisation, as did a further group who had poor language and literacy in the context of autistic disorder. Whitehouse and Bishop speculated as to the explanation for their results, and suggested that atypical lateralisation might relate either to severity of the language impairment, or to the specific profile of linguistic abilities. The data from the current study go against the former explanation: although the dyslexics in this study had poor reading ability relative to the non-dyslexic group, they did not have severe deficits. This was a result of using a predominantly student population, where reading ability needs to be adequate to deal with the demands of academia. Nonverbal ability was above average for both groups. Thus we find that even high-functioning individuals who have mild, circumscribed phonological problems affecting literacy, have a tendency to show reduced levels of left-hemisphere language compared to normal readers. This result is in direct opposition to predictions made by Leonard and Eckert (2008), who suggested that individuals with specific phonological problems were more likely to show an exaggeration of the normal pattern of left-lateralisation rather than reduction or reversal of left-hemisphere bias. Note, however, that their conclusions were based on data from structural rather than functional brain asymmetry.

At first glance, the current results seem to fit with Annett’s (1985) right shift theory, which postulated a single right shift (RS) gene with two allelic forms, one of which biases people to have left-hemisphere speech and right-handedness, with the other associated with lack of bias. The RS+ allele is seen as boosting language and phonological skills. However, our data do not fit Annett’s theory in detail, because it postulates a substantial effect of the RS+ gene, such that most people with bilateral or right hemisphere language would have two copies of the RS− allele, and so would be expected to show language or phonological deficits. More striking evidence against a strong link between atypical cerebral lateralisation and disorder comes from Knecht and colleagues (2001), who administered fTCD to a large sample of individuals, and compared those with left (*N* = 264), right (*N* = 31) and bilateral (*N* = 31) language on a wide range of behavioural measures, including intelligence, verbal fluency, mastery of foreign languages, and speed of processing. No differences were found. Any theory that regards atypical language lateralisation as *causing* language or literacy problems has difficulties accounting for such results.

We thus have a conundrum: most people with dyslexia have left-hemisphere language, and most people (from the general population) with bilateral or right-hemisphere language do not have language or literacy problems. Nevertheless, the distribution of LIs from the fTCD assessment is shifted closer to symmetry in a dyslexic group compared to the general population. Three classes of explanation seem plausible. The first account regards reduced lateralisation as a consequence rather than a cause of reading disability. The idea would be that as children develop reading skills, left-hemisphere reading systems develop. Evidence comes from an fMRI study of 113 dyslexic and 119 non-dyslexic children aged from 7 to 18 years by Shaywitz et al. (2007). These authors found an increase with age in lateralised activation of the anterior lateral occipito-temporal region during a reading task in the typical readers, but not in dyslexic readers. We argued against reduced lateralisation as a consequence of poor reading ability, because there was no correlation between performance on word generation and LI. Furthermore, normal lateralisation was seen by Whitehouse and Bishop (2008) in adult poor readers with autism. Nevertheless, we cannot exclude the possibility that our participants with dyslexia may have recruited different, less lateralised brain systems when doing word generation; it would be of interest to use fMRI with a comparable sample to test this idea.

A different type of explanation is given by a ‘multiple risk factors’ model, of a kind that has become popular in etiological accounts of developmental disorders. On this view, developmental dyslexia is a complex multifactorial disorder caused by the combined impact of a set of genetic and environmental risk factors, none of which is sufficient on its own to cause disorder (Bishop, 2006, 2009). Atypical cerebral lateralisation could be an index of one such factor, which assumes importance only when other risk factors are present. A ‘multiple risk factors’ account of the association between atypical laterality and dyslexia is consistent with the developmental instability theory of Yeo, Gangestad, and Thoma (2007). These authors regard atypical cerebral lateralisation as a marker of developmental instability, a phenomenon whereby a constellation of genetic and environmental factors can lead to increased noise in early developmental processes; in general, this will lead to more asymmetry in aspects of morphology that are usually symmetric, such as finger length or ear size. Where there is a general population bias to asymmetry, as with language representation in the brain, the predictions from the theory are more complicated. Yeo, Gangestad, Thoma, Shaw, and Repa (1997) argued that developmental instability can lead to either reduction or enhancement of typical asymmetry. Our current data, and those of Whitehouse and Bishop (2008), do not support the view that enhancement of left-hemisphere language laterality is seen in developmental language and literacy problems, but in other respects they fit with the developmental instability account. Indeed, it could be argued that the predictions from a developmental instability account depend crucially on how ‘developmental noise’ has its effects. If random noise is added to a process that generates a sidedness bias, the prediction is that the distribution will show increased variance, with an increased number of cases at either extreme. However, if the laterality distribution has a smaller contribution from a systematic biasing factor, and a greater contribution from randomness, then the distribution will be shifted toward symmetry, without an increase in variance, as was seen here.

A third theoretical account maintains that atypical language laterality in itself is not a risk factor, but the specific constellation of lateralised brain functions is important. We term this the ‘cognitive laterality profile’ hypothesis. It predicts that dyslexia might arise if both language and visuospatial functions are lateralised to the same hemisphere (leading to what Yeo et al. have termed ‘load imbalance’, sometimes also referred to as ‘functional crowding’), but not if the usual pattern of left-language and right-visuospatial is reversed. This might occur if there were competition for neural resources between functions. However, evidence against this comes from a study by Whitehouse and Bishop (2009) who found that in a sample from an elite academic institution, it was more common to find language and visuospatial skills represented in the same hemisphere than to see complete reversal of the normal pattern of lateralisation. Another possibility is a ‘ language laterality profile’ hypothesis, which maintains that risk of dyslexia increases if *within* the domain of language different skills are predominantly mediated in opposite hemispheres. A theory of this kind was put forward some years ago to explain language difficulties in Down syndrome (Elliott, Weeks, & Chua, 1994). We still know very little about the extent to which different language functions lateralise, and there is a tendency to treat ‘language’ as a monolithic skill. However, from the early days of Wada testing, it was clear that in some individuals, lateralisation varied according to the language test used, with some ‘bilateral’ individuals showing opposite patterns of laterality for naming versus serial ordering tasks (Milner, Branch, & Rasmussen, 1966). One can imagine that language processing might be less efficient if integrated function depended on combining information across hemispheres. Similar views have been put forward by Yeo et al. (1997) who suggested that for optimal functioning, modules that interact frequently should develop in close physical proximity. Further research is needed to identify paradigms beyond the word generation paradigm that can be used to study lateralisation of different components of language, and so to test this hypothesis.

The evidence used by Orton (1925) to argue for reduced cerebral lateralisation in dyslexia has not stood the test of time. Neither mirror-image confusions, nor non-right-handedness appear reliably associated with dyslexia (Bishop, 1990a), and Orton’s neurophysiological speculations were misguided. Nevertheless, now we have more direct and reliable means of assessing language laterality directly, Orton is shown to have been remarkably prescient. The association between atypical laterality and dyslexia is far from perfect, raising questions about the causal nature of the relationship, but at a group level, language lateralisation is indeed reduced in people with dyslexia compared to the general population. We now need research that integrates this perspective with current fMRI findings on connectivity among reading systems.

## 4 Methods

### 4.1 Participants

The two groups were matched on age, sex and nonverbal ability (see below). All participants gave written informed consent, and the project was approved by the Central University Research Ethics Committee of the University of Oxford.

### 4.2 Psychometric tests

Handedness was assessed using the Edinburgh Handedness Inventory (Oldfield, 1971), with scores ranging from −100 (completely left-handed) to 100 (completely right-handed). Nonverbal ability was assessed using the matrix reasoning subtest of the Wechsler Abbreviated Scale of Intelligence (WASI; Wechsler, 1999).

The York Adult Assessment (YAA; Hatcher, Snowling, & Griffiths, 2002) is a dyslexia assessment battery developed for the student population, which emphasises speed of reading and writing, rather than accuracy, and includes tasks that target phonological skills. It includes tests of nonsense passage reading, phonological awareness (Spoonerisms), writing speed, précis-writing and proof-reading. An in-house spelling test was substituted for the recommended WRAT 3 Spelling test because we had local normative data for this. For most of the YAA tasks, normative data are provided in the form of broad centile equivalents; these were used to derive standard scores with the means set at 100 and standard deviation 15, after Box–Cox transformations to optimise normality. Note that these norms are based on a student sample, rather than a general population sample. No normative data were provided for the proof reading task, so we calculated standard scores based on the mean and SD of the non-dyslexic group. A mean YAA score was also computed by averaging the subtest standard scores.

### 4.3 Apparatus

Bilateral blood flow was measured using a commercially-available Doppler ultrasonography device (DWL Multidop T2: manufacturer, DWL Elektronische Systeme, Singen, Germany), using two 2-MHz transducer probes mounted on a flexible headset. Visual stimuli (instructions, letters) were presented on a PC controlled by Presentation software (Neurobehavioral systems), which sent marker pulses to the Multidop system to mark the start of each epoch.

### 4.4 Stimuli

The word generation paradigm is described by Knecht et al. (1998). Verbally-produced words were recorded by the experimenter and the number of words per trial was calculated.

Data were analysed with the Average Software (Deppe et al., 1997), using the Autoedit function of Average 1.85. The period of interest was the interval from 10 to 18 s, based on previous research (Knecht et al., 2001). The LI is measured from the difference wave, and defined as the mean cerebral blood flow velocity in a 2 s window centred on the peak value in the period of interest. A positive LI indicates greater left than right hemisphere activation, and a negative LI indicates greater right than left hemisphere activation.

## Figures and Tables

**Table 1:** Mean and *SD* age and test scores for non-dyslexic and dyslexic groups.

	Non-dyslexic		Dyslexic	
	*N* = 30		*N* = 30	
	Mean	*SD*	Mean	*SD*
Age (yr)	22.4	3.4	24.2	4.4
Edinburgh handedness	68.5	40.0	51.8	53.4
Nonverbal *T*-score	59.4	6.3	56.7	6.0
Nonsense reading: errors	101.5	13.0	76.0	10.6
Nonsense reading: time	98.5	12.7	80.8	16.0
Spoonerisms, correct	92.6	7.5	81.3	11.7
Spoonerisms, time	103.9	10.6	89.1	14.4
Writing speed	113.4	27.0	87.7	19.9
Speeded spelling	112.7	28.9	83.1	18.0
Precis reading time	102.1	10.1	90.7	14.3
Precis writing	96.6	8.1	96.3	10.9
Precis content	105.7	9.7	93.1	10.1
Proof reading time	103.5	16.7	91.1	17.5
Proof reading errors	110.8	20.8	92.8	29.1
YAA average	103.7	8.9	87.4	8.2
